# The effect of quaternary ammonium polyethylenimine nanoparticles on bacterial adherence, cytotoxicity, and physical and mechanical properties of experimental dental composites

**DOI:** 10.1038/s41598-023-43851-y

**Published:** 2023-10-15

**Authors:** Grzegorz Chladek, Izabela Barszczewska-Rybarek, Marta Chrószcz-Porębska, Anna Mertas

**Affiliations:** 1 Faculty of Mechanical Engineering, Materials Research Laboratory, Silesian University of Technology, 18a Konarskiego Str., 41-100 Gliwice, Poland; 2 Department of Physical Chemistry and Technology of Polymers, Silesian University of Technology, 9 M. Strzody Str., 44-100 Gliwice, Poland; 3 Department of Microbiology and Immunology, Faculty of Medical Sciences in Zabrze, Medical University of Silesia in Katowice, 19 Jordana Str., 41-808 Zabrze, Poland

**Keywords:** Nanoparticles, Dental materials, Composite resin

## Abstract

A significant problem related to the functioning of resin-based composites for dental fillings is secondary or recurrent caries, which is the reason for the need for repeated treatment. The cross-linked quaternary ammonium polyethylenimine nanoparticles (QA-PEI-NPs) have been shown to be a promising antibacterial agent against different bacteria, including cariogenic ones. However, little is known about the properties of dental dimethacrylate polymer-based composites enriched with QA-PEI-NPs. This research was carried out on experimental composites based on bis-GMA/UDMA/TEGDMA matrix enriched with 0.5, 1, 1.5, 2 and 3 (wt%) QA-PEI-NPs and reinforced with two glass fillers. The cured composites were tested for their adherence of *Streptococcus Mutans* bacteria, cell viability (MTT assay) with 48 h and 10-days extracts , degree of conversion (DC), water sorption (WSO), and solubility (WSL), water contact angle (CA), flexural modulus (E), flexural strength (FS), compressive strength (CS), and Vickers microhardness (HV). The investigated materials have shown a complete reduction in bacteria adherence and satisfactory biocompatibility. The QA-PEI-NPs additive has no effect on the DC, VH, and E values. QA-PEI-NPs increased the CA (a favorable change), the WSO and WSL (unfavorable changes) and decreased flexural strength, and compressive strength (unfavorable changes). The changes mentioned were insignificant and acceptable for most composites, excluding the highest antibacterial filler content. Probably the reason for the deterioration of some properties was low compatibility between filler particles and the matrix; therefore, it is worth extending the research by surface modification of QA-PEI-NPs to achieve the optimum performance characteristics.

## Introduction

According to the latest data close to 39% of the world population have suffered from untreated dental caries in deciduous or permanent teeth and this percentage has increased extensively in the last 10 years^1,2^. Furthermore, nearly 60% of adolescents and more than 90% of the adult population have experienced dental caries^3^—in the first stage of treatment most of them get dental fillings made of photopolymerizable resin-based composites. Their matrixes are based on a mixture of dimethacrylate monomers such as bisphenol A glycerolate dimethacrylate (Bis-GMA), ethoxylated bisphenol A dimethacrylate (Bis-EMA), triethylene glycol dimethacrylate (TEGDMA) and/or urethane dimethacrylate (UDMA)^4^, which make it possible to develop materials characterized by advantageous functional features, including satisfactory aesthetic, physicochemical and mechanical properties^5–7^.

The most frequent problem that causes the replacement of 57% to 88% of resin-based composite fillings is secondary caries^8,9^. It is usually associated with the presence of a marginal gap induced primarily by polymerization shrinkage as well as the presence of porosities or other imperfections in the adaptation of material to tooth tissues^10,11^ with the simultaneous presence of pathogenic bacteria and products of their metabolism between restorations and teeth^12^. It is also believed that bacterial adhesion with the biofilm accumulation that occurs on the surface of composite restorations is related to the initiation of secondary caries^13,14^. Moreover, acid products of bacteria metabolism not only dissolve tooth minerals but may also lead to the degradation of composite restorations^14^. Another serious problem is the remaining caries caused by the imperfect removal of the tissues of infected teeth during treatment^15^. For these reasons, special attention has been focused on the development of new resin-composites with antibacterial properties to avoid colonization of the restorations’ surface and/or the tooth-restoration interfaces by cariogenic bacteria^16,17^. Different experimental strategies aimed at solving this problem have been considered. Antimicrobial agent release usually allows high local doses of antimicrobial agents to be obtained at specific sites and reduce the risk of systemic toxicity, but the durability of the effect is short and functional properties can often decrease. On the other hand, the contact-dependent strategy often has less or no adverse effects on mechanical properties, antibacterial activity is prolonged, but it is relatively weak with the risk of further reduction with surface biofouling^17^. In the last decade, much attention has been paid to dental composites enriched with antimicrobial nanoparticles and submicrometer-size particles such as silver^18^, zinc oxide^19^, cellulose nanocrystal/zinc oxide nanohybrids^20^, zinc-doped mesoporous silica nanoparticles^21^, silver sodium hydrogen zirconium phosphate^22^, titanium dioxide^23^ or chlorhexidine release systems^24,25^. Some works suggest the use of essential oils^26^. Other polymerizable compounds such as imidazole, chitosan loaded with dibasic calcium phosphate anhydrous, and chitosan particles show promising antimicrobial and biofunctional properties^27,28^. These solutions are characterized by a different level of success in laboratory tests, and the most frequently reported problems include decreased aesthetic properties^29^, cytotoxicity^30^, and decreased mechanical properties^31,32^.

Another nanomaterial considered as an antimicrobial filler for resin-based composites are cross-linked quaternary ammonium polyethylenimine nanoparticles (QA-PEI-NPs) in which the quaternary ammonium groups on the surface are responsible for antibacterial activity^33^. The antimicrobial activity of QA-PEI-NPs against standard Gram-positive (such as *Staphylococcus aureus*) and Gram-negative (such as *Escherichia coli*) strains and numerous other pathogenic bacteria strains has been proven^33–36^. It was also confirmed with diversified methodology that QA-PEI-NPs show activity against cariogenic bacteria *Streptococcus mutans* in in vitro tests^37–40^ and even against intraoral biofilm during an in vivo experiment^41^. However, little is known about other consequences of using QA-PEI-NPs as additives to resin-composite materials, because only in few studies modified commercially available resin-composite materials were investigated. Shvero et al.^38^ show that 2% of QA-PEI-NPs do not change the degree of conversion of modified composites. Beyth et al.^39^ introduced 1% (w/w) of QA-PEI-NPs into the flow and hybrid composite and registered a reduction in flexural strength (40%) for one of them. Beyth et al. and Yudovin-Farber et al.^34,42^ introduced up to 2 wt% of QA-PEI-NPs and reported no changes in cell viability after cytotoxicity tests. Barszczewska-Rybarek et al.^43^ conducted more complex investigations, where up to 2 wt% of QA-PEI-NPs was introduced into the resin mixture of 60 wt% bis-GMA and 40 wt% TEGDMA (no reinforcing fillers were used). They registered an increase in water solubility and contact angle, a decrease in flexural strength (57%) and impact resistance, however, antimicrobial and cytotoxicity were not tested.

On the basis of the above, QA-PEI-NPs have been shown to be promising antibacterial additives for dental photopolymerizable resin-based composites. Research conducted so far mainly examined the microbiological properties of modified commercial materials, while the availability of tests of mechanical or physicochemical properties is very limited. Previous tests were also carried out with the use of QA-PEI-NPs concentrations not exceeding 2% (w/w). It is also interesting to find out whether and how the introduction of reinforcing fillers can change some unfavorable consequences observed in the case of the QA-PEI-NPs—resin matrix system. Moreover, no previous work has examined the influence of QA-PEI-NPs on the properties of the composites, at the same time taking into account the biological, physicochemical, and mechanical properties, because in each of the available studies other commercially available composites were modified as starting material. As a result, the research conducted so far cannot be considered as a coherent whole for methodological reasons. In order to gain additional knowledge it is therefore necessary to conduct tests with the use of experimental model composite materials with a precisely known composition with a wider range of QA-PEI-NP concentrations. This article aims to provide for the lack of knowledge about the property relationships of dental dimethacrylate polymer-based composites enriched with QA-PEI-NPs. The research hypothesis was that QA-PEI-NPs introduction into experimental composites considered as dental restorative materials allows to obtain a reduction of adhered cariogenic bacteria on surfaces while maintaining the desired biofunctional properties. The null hypothesis was that the introduction of QA-PEI-NPs would not affect the mentioned properties of experimental composites.

## Materials and methods

### Synthesis of QA-PEI-NPs

The QA-PEI-NPs were synthesized with the method described by by Youdovin-Farber et al.^44^ with additional specifications described in details by Barszczewska-Rybarek^43^. The poly(2-ethyl-2-oxazoline) (PEtOx) (Sigma Aldrich, St. Louis, MO, USA) was dissolved in distilled water in a flask equipped with a magnetic stirrer, thermometer and condenser. Aqueous solution of hydrochloric acid (37 wt%, Acros Organics, Geel, Belgium) was added, and the reaction mixture was refluxed with the use of the oil bath. The mixture was cooled (room temperature) and unreacted hydrochloric acid and formed propionic acid were removed under reduced pressure. The residue acid was neutralized with sodium hydroxide (Chempur, Piekary Śl., Poland) solution in distilled water to pH 9–10. The obtained white PEI was recrystallized from distilled water, dissolved in methyl alcohol, purified by precipitating into ice-cooled diethyl ether, next filtrated and finally dried under reduced pressure. The solution of the obtained PEI in anhydrous ethanol (Stanlab, Lublin, Poland) was introduced into a flask equipped as previously described, the 1,5-dibromopentane (Acros Organics, Geel, Belgium) was introduced and the reaction was refluxed for 24 h. Next, the 1-bromooctane (Acros Organics, Geel, Belgium) was introduced and the mixture was refluxed again for 24 h and hydrobromic acid was neutralized with sodium bicarbonate (Chempur, Piekary Śl., Poland). For quaternization reaction the mixture was refluxed for 48 h after adding iodomethane and the resulting hydroiodic acid was neutralized with sodium bicarbonate under reflux. The obtained QA-PEI-NPs were purified by precipitation in distilled water, washing several times with hexane (Chempur, Piekary Śl., Poland) and distilled water, centrifuged, and finally lyophilized to dryness.

### Composite preparation

The matrix consisted of bisphenol A glycidyl methacrylate (bis-GMA), urethane-dimethacrylate (UDMA), and triethylene glycol dimethacrylate (TEGDMA) mixed at a weight ratio of 40:40:20, respectively, 0.4% (w/w) of camphorquinone as the photosensitizer, and 1% (w/w) of N,N-dimethylaminoethyl methacrylate (DMAEMA) as a photoaccelerator (all ingredients purchased form Sigma-Aldrich, St. Louis, MO, USA). The reinforcing fillers were two types of silanized barium borosilicate glass with a mean particle size of 2 μm (Schott AG, Landshut, Germany) and 0.7 µm (Esschem, Linwood, PA, USA) used at a weight ratio of 65:35, respectively.

The QA-PEI-NP nanoparticles were compounded at concentrations of 0.5, 1, 1.5, 2, 3% (w/w), and the masses necessary to prepare the composites were calculated according to the equation:

1 $${\mathrm{m}}_{\mathrm{QA}-\mathrm{PEI}-\mathrm{NP}}=\frac{{c}_{\mathrm{QA}-\mathrm{PEI}-\mathrm{NP} }\times {m}_{C}}{1- {c}_{\mathrm{QA}-\mathrm{PEI}-\mathrm{NP} }}$$

where m_QA-PEI-NP_ was the QA-PEI-NPs mass, g; c_QA-PEI-NP_ was the QA-PEI-NPs concentration, % (w/w); m_c_ was the matrix with glass fillers mass (constant), g.

The fillers were compounded in 50 mL glass beakers at room temperature in a darkroom equipped with a red LED lamp. All composites were prepared based on a 35 g portion of the matrix, and reinforcing fillers. The compounding was carried out gradually in standard portions of maximum 0.25 g of fillers (smaller for the 0.5% of QA-PEI-NPs or for the last portions of fillers) in the following order: QA-PEI-NPs, 0.7 µm glass, and 2 µm glass. Compounding was achieved by multiple, subsequent spreading and mixing of composition. The compounding process for a 35 g portion of composite took approximately 2 h to 2.5 h—longer time was required for higher concentrations due to increased viscosity. The prepared materials were placed under the pressure of 80 mbar for 2 h in a modified vacuum stirrer (Twister evolution, Renfert, Germany) to remove air bubbles created during compounding and finally were transferred to dark pharmacy jars, where they have been safely stored.

Photopolymerizations were carried out in teflon disc-like molds (degree of conversion (DC), compressive strength (CS), Vickers microhardness (HV)) and stainless steel molds (microbiological tests, water sorption (WSO), and solubility (WSL), water contact angle (CA), flexural modulus (E), flexural strength (FS)). The mold was placed on a microscope slide, material was packed in the mold, covered with 50 µm thick polyester foil and another microscope slide, that was pressed for 1 min to remove excess material. All samples were irradiated between two 50 W LED panels, each measuring 25 mm × 25 mm and equipped with 50 BRIDGELUX 45mil diodes characterized with optical wave length 440–450 nm (Fremont, USA). Irradiation time was 40 s at room temperature. After polymerization slides and foil were taken away, excess material was removed, samples were pushed out of molds, rinsed with distilled water and stored in distilled water in dark conditions at 37 °C for 24 h.

The experimental composite without the addition of QA-PEI-NPs was used as a control material, but to compare its microbiological properties with commercially available products, additional tests using Arkona Boston (AB) composite (Arkona Laboratory of Dental Pharmacology, Poland) were prepared. AB is composed of bis GMA, TEGDMA, UDMA, bis EMA, fillers measuring from 20 nm to 2 µm (barium borosilicate glass and silica) and CQ:DMAEMA photoactivating system.

Due to the influence of surface roughness on the results of microbiological tests (adherence of bacteria and cell viability assay), these samples were wet-ground with P500-grit abrasive paper to standardize the surface and rinsed with distilled water. The goal was to obtain comparable surface finishing with was controlled (Ra) with a Surtronic 25 contact profilometer (Taylor-Hobson, UK). On each surface (top and bottom), 3 measurements were made (radially), and the mean value was taken as the roughness value for sample^45^. The length of the measurement path was 0.8 mm and the range was 10 µm. The results were then subjected to statistical analyses to confirm that there were no significant differences between the groups..

### Adherence of bacteria

Bacterial adhesion tests were performed by incubating 10 mm × 1.5 mm (diameter × thickness) disc-shaped samples. Surfaces of all samples were wet-ground with P500-grit abrasive paper. All samples were placed for 18 h in 1 mL of *Streptococcus mutans* ATCC 33535 bacterial suspension ~ 5 × 10^6^ CFU/mL (CFU—colony forming units) at 37 °C. The samples were cleaned with sterile water and vortexed (1 min, 3000 rpm) in 1 mL of sterile water, 100 µL of the bacteria suspensions were serially diluted with 0.9% NaCl. 100 µL of these solutions and 100 µL of undiluted bacteria suspensions were seeded onto Columbia agar (bioMerieux, Marcy l’Etoille, France) with 5% sheep blood plates. The cultured plates were finally incubated at 37 °C for 24 h, and the number of bacteria that adhered to the surfaces was determined by counting the colonies.

### Cell viability assay (MTT assay)

#### Obtaining extracts of tested composites

Extracts of the tested composites were obtained according to the procedure in accordance with the EN ISO 10993-5:2009 standard. Samples of each of the materials tested were placed individually in the wells of a 24-well plate in a volume of 2 mL of culture medium identical in its composition to the medium used for the culture of fibroblasts of the L-929 line used in further studies. The prepared plates were incubated at 37 °C in an atmosphere of 5% CO_2_ for 2 or 10 days, thus obtaining 2-day and 10-day extracts. Under the same conditions as for the composite samples, the culture medium itself was also incubated in the wells of the plate as controls. Extracts and control media collected after incubation were stored at − 80 °C until tests were performed to assess the viability of L-929 cells.

#### Cell culture of the L-929 line

In in vitro studies, mouse fibroblasts from the L-929 line (NCTC clone 929) purchased from the American Type Culture Collection (Manassas, VA, USA) were used. Cell line L-929 (ATCC, catalog number CCL-1) consisted of subcutaneous connective tissue fibroblasts of mice of the C3H/An strain. ATCC formulated Eagle’s Minimum Essential Medium (EMEM) with 10% horse serum penicillin (100 IU/mL), and streptomycin (100 μg/mL) was used for L-929 cells. Cell culture was carried out in 25 cm^2^ polystyrene flasks for the cultivation of adherent cells (Nunc EasYFlasksTM NunclonTMDelta from Nunc A/S, Roskilde, Denmark). Cells were continuously grown in an MCO-17 AIC incubator from Sanyo (Japan), providing constant culture conditions (37 °C, 5% CO_2_ atmosphere at 100% relative humidity). Cells were passaged at 2–3 day intervals. For experimental studies, a suspension with a final density of 1 × 10^5^ cells/mL of medium was used. The density of the cell suspension was assessed by microscopy using a Burker chamber.

#### Evaluation of the viability of L-929 cells contacted with the extracts of the tested composites

The cytotoxicity assessment of the tested composites was carried out according to the recommendations of the EN ISO 10993-5 standard^46^. Model L-929 cells (mouse fibroblasts) under in vitro culture conditions were contacted for 24 h with undiluted extracts. After 24 h of incubation, cell viability was assessed using the bromo-3-[4,5-dimethylthiazol-2-yl]-2,5-diphenyltetrazolium assay (MTT assay). In this test, the measurement of mitochondrial dehydrogenase activity made it possible to determine the percentage of live cells in cultures contacted with a specific extract, and thus to determine the cytotoxicity of the tested composites. According to the recommendations of the EN ISO 10993-5 standard, a reduction in the viability of cells contacted with the extracts tested by more than 30% as compared to the control cell culture (viability below 70%) was considered a cytotoxic effect.

Wells of a 96-well microplate were dispensed with 100 µL of L-929 cell suspension at a density of 1 × 10^5^ cells/mL (10,000 cells/well) in RPMI 1640 medium with 10% FBS, penicillin (100 IU/mL) and streptomycin (100 μg/mL). After 24 h of incubation at 37 °C in an atmosphere containing 5% CO_2_ and 100% relative humidity, the supernatants were removed and 100 μL of undiluted extract of a specific test preparation or medium after incubation conducted in parallel with obtaining extracts. The control culture consisted of cells contacted with fresh culture medium. After 24 h of incubation at 37 °C in an atmosphere of 5% CO_2_ and 100% relative humidity, MTT solution at a final concentration of 1.1 mM in fresh culture medium was dispensed into each well after removal of the culture medium. After 3 h of incubation at 37 °C in 5% CO_2_ at constant relative humidity, the supernatants were removed and 200 µL of DMSO was added to the test and control cultures to extract MTT formazan. After 20 min, 150 μL of the solution was taken from each well and its absorbance was determined at 550 nm using the Eon automatic plate reader (BioTek Instruments, Winooski, VT, USA). The intensity of the violet color of the solution was directly proportional to the amount of formazan formed and thus the number of viable cells.

Cell viability (%) was calculated using the following formula:

2 $$Cell \, viability=\frac{Ab}{Ak} \times 100\%$$

where: Ab—the absorbance of the test sample, Ak—the absorbance of the control.

### Degree of conversion

The *DC* was determined from the spectra obtained with the Fourier Transform Infrared Spectrometer (Spectrum Two, Perkin-Elmer, Waltham, MA, USA). Samples were analyzed in the form of KBr pellets with 128 scans at a resolution of 1 cm^−1^. The DC was calculated from the decrease of absorption band at 1637 cm^−1^ referring to the C=C stretching vibration (A_C=C_), in relation to the peak at 1608 cm^−1^, assigned to the aromatic stretching vibrations (A_Ar_) with the following equation^47^:

3 $$DC \left(\%\right)= 1-\frac{{\left(\frac{{A}_{C=C}}{{A}_{Ar}}\right)}_{after \, polymerization}}{{\left(\frac{{A}_{C=C}}{{A}_{Ar}}\right)}_{before \, polymerization}}\times 100\%$$

### Vickers hardness

Five cylindrical samples (6 mm in diameter and 3 mm in height) were prepared from each composite. Five indentations were made on each sample, and average values are denoted as hardness. Measurements were made at a 0,4905 N (0.05 kgf) load and a loading time of 15 s^48^ (Future-Tech FM-700 microhardness tester, Future-Tech Corp, Tokyo, Japan) and Vickers hardness was calculated automatically based on the average length of the diagonal left by the indenter.

### Compressive strength

Ten cylindrical samples (4 mm in diameter and 8 mm in height) were prepared from each composite, but according to the recommendation of Galvão et al.^49^ they were additionally light-irradiated on lateral surfaces after being removed from molds. The tests were carried out with a cross head speed of 0.5 mm/min at a universal testing machine (Zwick Z020 GmbH & Com, Ulm, Germany), and the CS values were calculated according to the equation:

4 $$\mathrm{CS}=\frac{F}{\mathrm{A}}$$

where: CS—the compressive strength, MPa; F—force at fracture, N; A—the initial cross-sectional area, mm^2^.

### Flexural strength

Three-point bending tests were carried out using a universal testing machine in accordance with the ISO 4049 standard^50^. Ten bar samples 25 mm × 2 mm × 2 mm (length × width × thickness) were prepared from each material. The cross-head speed was 0.75 mm/min and the distance between the supports was 20 mm. FS strength and E were calculated with the following formulas:

5 $$FS=\frac{3\mathrm{Pl}}{{2bh}^{2}}$$

6 $$E=\frac{{P}_{1}{l}^{3}}{{4bh}^{3}\delta }$$

where: l—the distance between the supports, mm; b and h—the width and height, mm; P—the maximal force, N; P_1_—the load at the chosen point at the elastic region of the stress–strain plot, kN; δ—the deflection in P_1_.

### Sorption and solubility

The test was carried out according to ISO 4049^50^. Five samples of each material (measuring 15 mm in diameter and 1 mm in height) were dried inside desiccators with freshly dried silica gel at 37 ± 1 °C and weighed daily (Analytic Scale AS 60/220.X2.PLUS, Radwag, Poland) with an accuracy of 0.1 mg to achieve a constant value of the mass m_1_ (daily changes < 0.1 mg). Samples were placed separately in 10 mL of distilled water for 7 days at 37 ± 1 °C and rapidly dried from visible moisture with filter paper, m_2_ mass values were registered, and the drying was repeated to constant mass registers as m_3_. The WSO and the WSL were calculated according to the following formulas:

7 $${\text{WSO}} = \frac{{{\text{m}}_{{2}} - {\text{m}}_{{3}} }}{{\text{V}}}$$

8 $${\text{WSL}} = \frac{{{\text{m}}_{{1}} - {\text{m}}_{{3}} }}{{\text{V}}}$$

where, m_l_—the initial mass of the dried sample, µg; m_2_—the mass after storing, µg, m_3_—the mass after the second drying, µg; and V—the volume of the sample after the first drying, mm^3^.

### Water contact angle

The five polymerized composites were tested for water contact angle (CA) using a goniometer (OCA 15EC, Data Physics, Filderstadt, Germany). Deionized water (4 µL) was dropped onto the tested surface via the sessile drop method.

### Statistical analysis

Statistical analysis was performed using the PQStat ver. 1.6.6.204 (PQStat Software, Poland). The residuals distributions were tested with Shapiro–Wilk, the equality of variances was tested with the Levene test and one-way ANOVA with a possible F * correction (Brown-Forsythe) and Tukey HSD post hoc tests were used (for all tests α = 0.05). The results of microbiological tests were statistically evaluated using non-parametric Kruskal–Wallis test (α = 0.05) with Dunn-Bonferroni post hoc test and Mann–Whitney U test .

## Results

**Figure 1:**
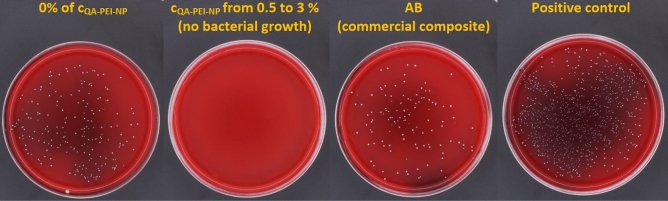
Representative images presenting the results of the *Streptococcus mutans* ATCC 33535 adherence test—cultured plates after incubation with undiluted bacteria suspensions.

The number of live *S. mutans* bacteria adhered to the surfaces decreased significantly (p < 0.0001) after the introduction of QA-PEI-NPs (Table 1, Fig. 1). For composites with QA-PEI-NPs no adhered bacteria were observed, for control composite material the average value was 17.03 × 10^2^ CFU/mL while for the commercial composite (AB) the average value was 11.56 × 10^2^ CFU/mL and the registered differences were not statistically significant (Supplementary Table 1) .

**Table 1:** The number of *S. mutans* bacteria adhered to composites’ surface and viability of L-929 cells after 24 h of incubation with the 2-days and 10 days extracts.

c_QA-PEI-NP_, %	Number of adhered bacteria, × 10^2^ CFU/mL		Viability of L-929 cells, %			
	(p < 0.0001)		2 days (p = 0.2795)		10 days (p = 0.0145)	
	AV	SD	AV	SD	AV	SD
0 (control)	17.03^a^	3.23	89.6	3.6	91.1^a^	3.2
0.5	No adhered bacteria^b^		91.0	5.5	84.7^a,b^	2.2
1	No adhered bacteria^b^		88.7	3.3	87.7^a,b^	3.1
1.5	No adhered bacteria^b^		87.0	2.0	82.1^a,b^	1.9
2	No adhered bacteria^b^		86.7	1.2	83.4^a,b^	3.8
3	No adhered bacteria^b^		82.2	5.2	78.5^b^	3.7

The different lowercase letters (a–b) for column show significantly different results at the level of p ≤ 0.05.

*AV* average value, *SD* standard deviation, *CFU* colony forming units.

**Figure 2:**
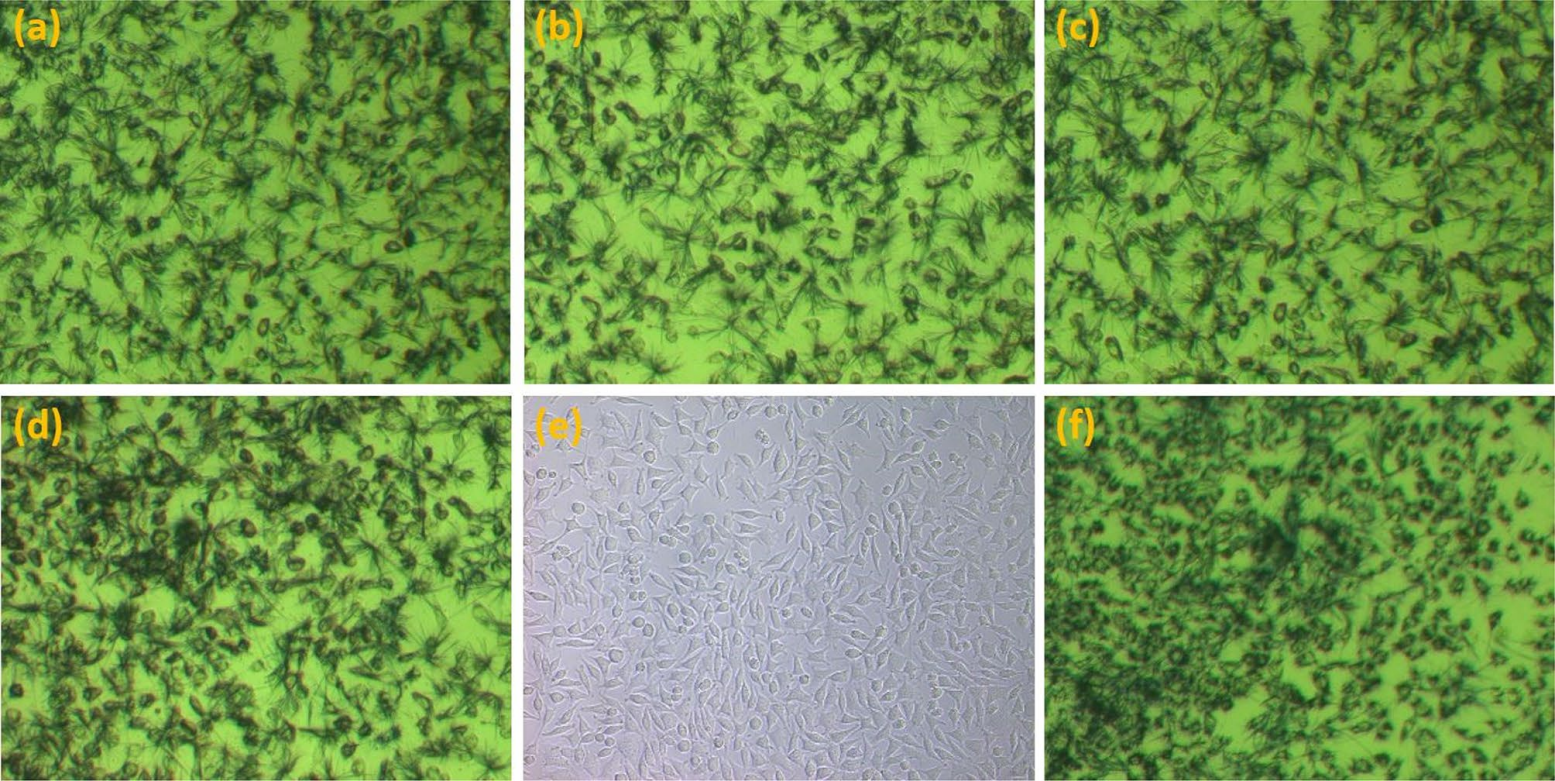
Representative images of L-929 line cells after 24 h incubation with undiluted 2 days (**a**) and 10 days (**b**) extracts of the experimental control composite (0% of QA-PEI-NP) after MTT assay, 2 days (**c**) and 10 days (**d**) extracts of the experimental composite containing 3% of QA-PEI-NP after MTT assay, control culture of adhered L-929 cells before MTT test (**e**) and control culture of adhered L-929 cells after MTT test (**f**).

**Figure 3:**
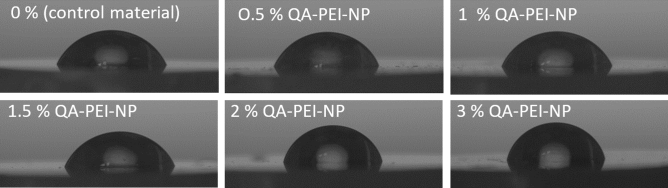
Representative images of deionized water droplets on the composite surfaces obtained from the goniometry camera.

The mean viability values of L-929 cells for the extracts of the tested materials are presented in Table 1 and Fig. 2 (the detailed results are summarized in Supplementary Table 2). For 2-day extracts, there was no statistically significant difference (p = 0.2795) between the results obtained for the control composite (89.6%) and the experimental composites, however, for 3% QA-PEI-NPs the average viability value was lower than for other materials (82.2%). For 10 days, the extracts showed a statistically significant decrease (p = 0.0145) in viability and the post hoc test confirmed that compared to control material (cell viability was 91.1%) cell viability decreased for 3% QA-PEI-NPs (p = 0.0118) for which the lowest value was observed (78.5%). The reduction in viability of L-929 cells due to the extension of the time to obtain the extract was registered for materials enriched with QA-PEI-NPs; however, it was not statistically significant (p > 0.05). An additional experiment (Supplementary Table 3) confirmed that there were no statistically significant differences in cell viability for the control composite and the commercially available product. Representative microscopic images of L-929 cells obtained for the experimental preparation compared to control cultures of adhered L-929 cells are presented in Fig. 3.

The HV of composites ranged from 47.4 HV0.05 to 50.3 HV0.05 and there were no statistically significant differences (p = 0.052) between average values (Table 2).

**Table 2:** Vickers hardness, compressive strength, flexural strength and flexural modulus after the introduction of QA-PEI-NPs.

c_QA-PEI-NP_, %	HV0.05		CS, MPa		FS, MPa		E, GPa	
	(p = 0.052)		(p < 0.0001)		(p = 0.0001)		(p = 0.169)	
	AV	SD	AV	SD	AV	SD	AV	SD
0	48.8	0.8	317.7^a^	13.1	100.1^a^	8.7	7.3	0.4
0.5	48.7	1.3	309.9^a^	9.6	91.8^a^	13.3	7.6	0.5
1	49.3	1.1	273.6^c^	9.2	90.9^a^	11.9	7.8	0.5
1.5	48.6	0.8	250.5^c,d^	20.2	89.4^a^	3.8	7.7	0.5
2	50.3	1.6	234.0^d,e^	20.5	85.3^b^	7.7	7.6	0.7
3	47.4	1.1	219.5^e^	14.3	78.0^b^	8.2	7.4	0.5

The different lowercase letters (a–e) for a column show significantly different results at the level of p ≤ 0.05.

*AV* average value, *SD* standard deviation.

The composites’ CS ranged from 219.5 MPa to 317.7 MPa (Table 2) and a statistically significant (p < 0.0001) decrease came with increasing QA-PEI-NPs concentrations. For composites with QA-PEI-NPs concentrations starting from 1% the CS values were significantly lower than for the control material (from p = 0.0001), however, there were no significant differences between materials enriched with 1.5% to 2% and 2% to 3% QA-PEI-NPs (p > 0.05).

The FS of composites ranged from 78.0 MPa to 100.1 MPa (Table 2) and decreased statistically significantly (p < 0.0001) with the increasing QA-PEI-NPs content. Statistically significant decreases were registered for composites enriched with 2% (p = 0.0008) and 3% (p = 0.0002) of QA-PEI-NPs. The E values ranged from 7.3 to 7.8 GPa and there were no statistically significant (p = 0.169) differences between the average values.

The DC of composites ranged from 59.8% to 64.2% and there were no statistically significant (p = 0.9451) differences between average values (Table 3).

**Table 3:** The degree of conversion, water contact angle, sorption and solubility of composites after the introduction of QA-PEI-NPs. *

c_QA-PEI-NP_, %	DC, %		WSO, µg/mm^3^		WSL, µg/mm^3^		CA, °	
	(p = 0.9451)		(p < 0.0001)		(p = 0.002)		(p = 0.0017)	
	AV	SD	AV	SD	AV	SD	AV	SD
0	63.6	8.9	20.0^a^	1.2	2.6^a^	0.2	73.0^a^	1.8
0.5	64.2	7.4	22.0^a,b^	2.2	3.7^a^	0.7	72.8^a^	1.2
1	61.3	5.8	22.3^a,b^	0.3	3.7^a^	0.6	73.4^a^	2.1
1.5	60.1	9.0	23.7^b,c^	0.6	4.0^a,b^	0.6	74.5^a,b^	1.8
2	59.8	8.8	23.9^b,c^	0.9	3.9^a,b^	0.3	76.6^a,b^	2.2
3	60.9	4.8	25.1^c^	0.9	5.6^b^	1.7	77.9^b^	1.6

The different lowercase letters (a–c) for column show significantly different results at the level of p ≤ 0.05.

*AV* average value, *SD* standard deviation.

The composites’ WSO ranged from 20.0 µg/mm^3^ to 25.1 µg/mm^3^ and increased with the increasing QA-PEI-NPs content (p < 0.0001) (Table 3). Statistically significant differences in comparison to control material were registered for the concentration of 1.5% QA-PEI-NPs (p = 0.0019, 18% increase), 2% QA-PEI-NPs (p = 0.001, 18% increase), and for the highest content (p = 0.0001, the increase was 25%). For most of the experimental composites the average WSO values did not differ statistically significantly, but statistically significant differences were registered for 3% QA-PEI-NPs in comparison to 0.5 QA-PEI-NPs (p = 0.0119) and 1 QA-PEI-NPs (p = 0.0223). The composites’ WSL ranged from 2.6 µg/mm^3^ to 5.6 µg/mm^3^. The increasing QA-PEI-NP content caused a gradual increase in WSL values (p = 0.0017), however, differences were statistically significant only for the highest QA-PEI-NP concentration where there was an abrupt increase of 119% compared to the control material. The statistically significant differences were registered for the control material, 0.5% QA-PEI-NPs and 1% QA-PEI-NPs vs. 3% QA-PEI-NPs (p = 0.0006, p = 0.0364, p = 0.465, respectively).

The composites’ CA ranged from 73.0° to 77.9°. Increasing QA-PEI-NP concentration caused a gradual increase in CA values (p = 0.0017). Differences were statistically significant for the highest QA-PEI-NP concentration and control material (p = 0.0088, the increase was 7%), 0.5% QA-PEI-NPs (p = 0.0059) and 1% QA-PEI-NPs (p = 0.02) composites.

## Discussion

The development of antibacterial resin-based composites is one of the most important pathways of investigations regarding direct dental filling materials. In this study, QA-PEI-NPs synthesized with the previously described method^43,51^ and characterized by an average size of 151 nm which was reported as optimal^43^ were introduced into a representative bis-GMA-TEGDAM-UDMA matrix, finally compounded with two glass reinforcing fillers^52^.

The antimicrobial action of QA-PEI-NPs against cariogenic bacteria was recorded in previous in vitro studies. Beyth et al.^39^ and Shvero et al.^38^ found that commercially available flow and hybrid composites modified with 1% of QA-PEI-NPs present durable antibacterial activity against *S. mutans* in a direct contact test and strong activity during 24 h contact confirmed by SEM investigations. Similar results were obtained with an analogous methodology for additional modified commercial composites^37,40^. In these investigations, only formulations that contained 1% and 2% were tested. In our study, strong antibacterial activity against *S. mutans* was also confirmed for a lower concentration (0.5%) with another methodology, because the current experiment was based on the adherence of bacteria cells to the samples’ surfaces. Due to the nature of the test, attention was paid to the finishing of the samples’ surface, because roughness is related to bacterial adherence to the composites^53,54^. All samples were ground which selected abrasive sandpaper which gave a roughness measured during quality control of ~ 0.35 µm (from 0.27 to 0.4 µm) which corresponds to the typical values after finishing the fillings under clinical conditions, that range from 0.1 µm to 0.6 µm (usually from 0.25 µm to 0.4 µm)^55–59^. The antibacterial action of the used NPs is related to the properties of positively charged quaternary ammonium groups of QA-PEI, which interact with negatively charged bacterial cell walls disrupting its electrical balance, which finally leads to a decrease in its osmoregulatory and physiological roles of the membrane resulting in cell death^60^. Introducing this polymeric additive in the form on NPs is advantageous, because of its specific surface being exceptionally large, so low concentrations can be used to achieve antimicrobial activity^61^. However, the condition for success is to obtain a homogeneous distribution of the antimicrobial filler^62^ that should be close to 1 NP/µm^2^ to ensure its contact leading to activity against bacteria^38^. Our research has shown that at a concentration of QA-PEI-NPs lower than previously used, it is still possible to achieve a sufficient distribution of particles to prevent cariogenic bacteria cells from surviving on the surface. Some limitation indicating the need for caution in interpreting the results may be the fact that adsorption of saliva ingredients to the surface and the creation of biofilms on the fillings may potentially cover the antibacterial groups of QA-PEI-NPs, regardless of the results of an in vivo experiment which suggests, that after 4 h of biofilm creation, the antibacterial action is still effective^41^.

Biocompatibility tests showed that cell viability for incubation times exceeded 70%, so none of the materials showed cytotoxic properties after two as well as after ten days of extraction^46^. Furthermore, there were no differences in cell viability after two days of incubation for all materials, which stay consistent with previous cytotoxicity investigations conducted with different methodologies and cell lines for composites with up to 2% of QA-PEI-NPs^34,42^. After a ten-day extraction of the samples containing the highest concentration, a decrease of 14% in cell viability was observed compared to the control material. Other studies reported that QA-PEI-NP suspensions reduced cell viability in cytotoxicity assay, but cytotoxicity occurred only at concentrations a few times higher than necessary to obtain an antibacterial effect^63^. This stays in accordance with our results. Yudovin-Farber et al.^44^ suggested that the reduction of cell viability in the cytotoxicity test against mammalian cells caused by QA-PEI-NPs is related to a strong positive charge of the nanoparticles, so its action is analogous to the one previously described for prokaryotic cells. However, it is emphasized that the mechanism of binding to cell walls causing membrane disruption through direct interactions or through reactive oxygen species production is much less toxic in mammalian cells than in bacteria, because the first ones may phagocytize NPs, and degrade them by lysosomal fusion to a certain degree with finally reduced toxicity^63,64^. This may be the reason why, even after a longer than typical time of obtaining extracts, the reduction of fibroblast cell viability even for the highest concentration of QA-PEI NPs associated with their presence on the surface and/or their leaching was still small.

The degree of conversion of double bonds is an important feature of dental composites based on dimethacrylate monomers due to the potential risk of biological responses of pulp tissue related to the release of monomers^65^ as well as the influence on mechanical and physicochemical properties^66^. Our results are in agreement with the earlier findings, where 2% of the QA-PEI-NPs did not significantly change the DC values of the modified commercial composites^38^ and the Bis-GMA/TEGDMA matrix^43^. The DC values were also typical for formulations based on similar dimethacrylate systems^67^. As light passes through the solid material with increasing density, such as a filled composite, its intensity decreases^68^. Moreover, the composite ingredients such a fillers scatter and absorb light more effective than the matrix, decreasing the power density with distance from the sample surface and finally decreasing the DC values^69^.Increased scattering in the composites also occur when filler particle size is from one-half to close to that of the curing light wavelength^70^. Our earlier work related to the characterization of the QA-PEI-NPs showed only a small percentage of particles of this size in the synthetized powder^43^ and the concentrations of QA-PEI-NPs were low (max. 3%) in comparison to those of other fillers content, so their influence on the additional scattering or absorption of light was not sufficient to affect significantly the DC of composites. It cannot be ruled out that an increase in the content of QA-PEI-NPs could lead to such changes. The potential reduction in DC after introducing new antimicrobial fillers has been recognized to be correlated with decrease in microhardness^22,71^, which is consistent with our research, as both the DC and hardness values did not change significantly. In this context, microhardness measurements confirm the preservation of beneficial properties of the polymer matrix. On the other hand, the introduction of ceramic fillers usually increases the hardness due to their characteristics and the high interfacial shear strength between the nanofiller and the resin matrix, which increased the resistance to localized plastic deformation^72^. However, the nanoparticles used in this work, due to the comparable hardness of antimicrobial polymers and typical polymeric matrixes of dental composites^73,74^ cannot lead to an increase in hardness. In this context, the results achieved were favorable. In addition, stable microhardness values should be considered beneficial because a reduction in hardness can lead to increased wear^75,76^ and swallowing more friction products with chewed food and saliva.

Three mechanical properties on the macroscale were analyzed, the compressive, flexural strength, and flexural modulus. Compressive forces are generated during mastication, especially when composite fillings replace a large volume of tooth tissues^77^, while flexural forces are particularly important in stress-bearing cavities for restoration classes I, II, and IV^78^. The decrease in CS values was significant for materials enriched with QA-PEI-NP excluding one that contained 0.5% filler; however, for concentrations of 1% it was 14%, while for 3% it was 31%. The recommended values of CS are comparable with the plastic limit of tooth tissues^49,79^, whose average values are up to 170 MPa for dentine and up to 225 MPa for enamel^80^. This indicates that up to 1.5% of QA-PEI-NP CS values were fully satisfactory, but starting from 2% they may not allow the full use of the load-bearing capacity of tooth tissues in some cases. The effect of QA-PEI-NPs on CS composites was not investigated in the past; however, the introduction of metallic or ceramic antibacterial particles gives diversified results, from no effect even at high contents for cubic-shaped silver-enriched ceramic particles^22^ to increasing at low and decreasing at higher concentrations of metallic and ceramic NPs^81–83^. FS values systematically decreased with increasing concentration of QA-PEI-NP; however, statistically significant reductions of 15% and 22% were recorded for 2% and 3%, respectively. For the highest content of QA-PEI-NP, the registered values were below the limit of 80 MPa recommended by the ISO 4049 standard for restorations of the occlusal surfaces^50^. Beyth et al.^39^ reported that 1% of QA-PEI-NP resulted in a reduction in FS by approximately 40% for a composite based on the bis-GMA/TEGDMA matrix and 47% of reinforcing fillers, but do not have a significant influence on the commercial material based on the bis-GMA/UDMA/TEGDMA matrix reinforced with 60% fillers. Barszczewska-Rybarek et al.^43^ also reported a significant reduction of FS values (up to 57%) for Bis-GMA/TEGDMA resin after introducing 0.5% to 2% of QA-PEI-NPs. After considering their own and Beyth et al.^39^ results, the authors suggested that matrixes containing UDMA due to participation in strong hydrogen bonding may allow one to create formulations that are less sensitive to flexural stress after introducing new particles. Our results support this concept because decrease in FS was not significant up to 1.5% of QA-PEI-NPs. However, it does not explain still large FS and CS changes at relatively low concentrations of QA-PEI-NPs, because numerous types of particles with antimicrobial properties introduced into similar matrixes do not degrade mechanical properties at such content. Stencel et al.^22^ showed a reduction in FS after compounding a 16% cubic-shaped ceramic filler, Tavassoli Hojati et al.^83^ introduced up to 5% of ZnO-NPs into a bis-GMA/UDMA/TEGDMA-based composite, while Brandão et al.^71^ compounded up to 10% of ZnO-NPs into a bis-GMA/TEGDMA-based material, and both did not register significant changes in FS. The reduction of the mechanical properties on a macroscale after using QA-PEI-NPs even at low concentrations may suggest weak compatibility of QA-PEI-NPs with the resin phase, which results from poor wettability of particles by matrix components and/or adhesion of phases. Weak interactions between these ingredients may result in creation of voids around the particles, which are structural defects. They may expand when aload is applied and act as stress concentrators, leading to a decrease in CS and FS. This shows that further investigations should take into account physical or chemical surface modifications of QA-PEI-NPs to eliminate this problem^84^.

The clinically acceptable values of the flexural modulus can vary, with a relatively lower modulus to flex with the teeth for cervical cavities or higher to withstand the occlusal forces for posterior composites^85^. The E values of the composites studied did not change significantly due to the addition of QA-PEI-NPs, which remain in accordance with provirus reports^39,43^ and can be considered an advantage because it indicates that there were no significant changes in the molecular structure of the polymer network such as an increase in the loop number that could cause a decrease in E values without a decrease in the DC^66^.

Water sorption and solubility are other key physicochemical properties of dental composites due to their potential influence on biocompatibility related to the washout of ingredients and being often associated with the stability of dimensions or mechanical properties caused by liquid uptake^86^. The registered WSO values for all samples were lower than 40 μg/mm^3^, however WSL values for 2 samples with 3% of QA-PEI-NPs exceeded 7.5 μg/mm^3^, so these materials did not meet the requirements of the ISO standard^50^. The increase in WSO/WSL has been reported for materials containing nanoparticles^69,87^, including QA-PEI-NPs^43^. Matrix properties such as a decrease in DC are often mentioned in the context of the relationship with the increase in WSO/WSL^88,89^, however in this study no differences in DC were observed, which suggest another cause of changes. Increased WSO and WSL can be another factor indicating low compatibility between filler particles and the matrix, because water can migrate and accumulate at the matrix-filler particles and/or their aggregation interface, which may explain especially larger changes for higher filler concentrations^7,22^. Large surface area, typical for the NPs, may also favor degradation processes such as hydrolysis of filler and lead to particle loss^69^, which can be additionally accelerated due to the increased contact of water with particles at the matrix-filler interface when their compatibility is low. Water can migrate into the filler-matrix interface and into free spaces when particles were lost, increasing the WSO. An increase in the WSL values may also be related to the QA-PEI-NPs because of possible weaker interactions between filler particles and water molecules in comparison to those between QA-PEI-NPs and matrix. The leaching of particles may also increase the contact area of the matrix with the water, which intensified the leaching process of different components.

The water contact angle is discussed due to its influence on plaque formation because materials with high CA demonstrate less bacterial adherence^90^. The results showed that the CA of the tested surfaces increased to 6% for the highest concentration of QA-PEI-NPs (other changes were insignificant), however, all values obtained were less than 90°, so the character of the surfaces was still hydrophilic^91^. The registered increase in CA can be caused by the presence of hydrophobic N-octyl substituents in QA-PEI-NPs^43^.

## Conclusions

The article provided new information on the properties of model composites based on the bis-GMA/UDMA/TEGDMA matrix reinforced with glass fillers and enriched with QA-PEI-NPs. The results showed that the compounding from 0.5 to 3% of QA-PEI-NPs allows to obtain high antimicrobial effectiveness and satisfactory biocompatibility. The QA-PEI-NPs introductions have no effect on the degree of conversion DC, Vickers microhardness, and flexural modulus’ values and caused a favorable increase in contact angle. On the other hand, QA-PEI-NPs increased water sorption and solubility, decreased flexural strength, and compressive strength. The mentioned changes were insignificant and acceptable for most experimental composites, excluding the highest antibacterial filler content. Probably the reason for the deterioration of some properties was low compatibility between filler particles and the matrix; therefore, it is worth extending the research by surface modification of QA-PEI-NPs to achieve optimum performance characteristics.

### Supplementary Information

Supplementary Tables.

## Data Availability

The datasets used and/or analyses during the current study available from the corresponding author on reasonable request.
