# Supplementary material for: The effect of quaternary ammonium polyethylenimine nanoparticles on bacterial adherence, cytotoxicity, and physical and mechanical properties of experimental dental composites
Source: Sci Rep. 2023 Oct 15;13:17497. doi: 10.1038/s41598-023-43851-y (PMC10577145; doi:10.1038/s41598-023-43851-y)
Supplement: Supplementary file 1 — Supplementary Tables. [file 41598_2023_43851_MOESM1_ESM.pdf]

Supplementary Table 1. The adherence test results (*S. mutans*) for experimental control material and commercial composite (AB).

	Number of adhered bacteria, $\times 10^2$ CFU/mL							
$c_{QA\text{-}PEI\text{-}NP}$, % / abbreviation	0	0.5	1	1.5	2	3	AB	Positive control
	15.1	0	0	0	0	0	13.6	Control
	14.6	0	0	0	0	0	13.5	131.1
	12.5	0	0	0	0	0	12.5	120.4
	21.1	0	0	0	0	0	10.6	116.9
	20.9	0	0	0	0	0	10.0	115.0
	18.0	0	0	0	0	0	9.2	93.0
AV	17.0	0	0	0	0	0	11.6	110.1
SD	32.3	0	0	0	0	0	17.2	16.3

Supplementary Table 2. The absorbance values and viability of L-929 cells after 24 h of incubation with the 2-days and 10 days extracts.

	Absorbance, λ = 550 nm*,							Cell Viability Assay test, %						
$c_{QA\text{-}PEI\text{-}NP}$, %	0	0.5	1	1.5	2	3	CM**	0	0.5	1	1.5	2	3	CM**
2 days extracts	0.210	0.192	0.201	0.199	0.199	0.181	0.253	89.7	82	85.9	85	85	77.4	108.1
	0.217	0.227	0.201	0.199	0.205	0.182	0.210	92.7	97	85.9	85	87.6	77.8	89.7
	0.196	0.218	0.208	0.208	0.206	0.195	0.237	83.8	93.2	88.9	88.9	88	83.3	101.3
	0.216	0.215	0.220	0.208	0.202	0.211	0.280	92.3	91.9	94	88.9	86.3	90.2	119.6
10 days extracts	0.202	0.200	0.212	0.185	0.204	0.177	0.227	86.3	85.5	90.6	79	87.2	75.6	97.0
	0.214	0.190	0.197	0.193	0.204	0.177	0.215	91.4	81.2	84.2	82.5	87.2	75.6	91.9
	0.214	0.204	0.213	0.193	0.185	0.183	0.210	91.4	87.2	91	82.5	79	78.2	89.7
	0.223	0.199	0.199	0.197	0.188	0.198	0.247	95.3	85	85	84.2	80.3	84.6	105.6

*The mean value of the absorbance of the control sample (cells contacted with fresh culture medium) from six replicates was *0.234; ** CM - culture medium incubated without composite

Supplementary Table 3. Absorbance values and viability of L-929 cells after 24 h of incubation with the 2-days and 10 days extracts of experimental control (0% of QA-PEI-NPs) and commercial composite (AB).

Time of extracting	Absorbance, λ = 550 nm*			Cell Viability Assay test, %		
2 days	0 %	AB	CM**	0 %	AB	CM**
	1.117	1.22	1.235	77.5	84.7	85.7
	1.167	1.18	1.156	81	81.9	80.2
	1.189	1.174	1.194	82.5	81.5	82.8
	1.175	1.238	1.239	81.5	85.9	86
AV				80.6	83.5	83.7
SD				1.9	1.9	2.4
10 days	1.197	1.144	1.399	83.1	79.4	97.1
	1.215	1.253	1.246	84.3	87.0	86.5
	1.224	1.232	1.237	94.9	85.5	85.8
	1.161	1.312	1.16	80.6	91	80.5
AV				83.2	85.73	87.5
SD				1.7	4.2	6.0

*The mean value of the absorbance of the control sample (cells contacted with fresh culture medium) from six replicates was *1,441; ** CM - culture medium incubated without composite; cell viability assay test results: 0% vs AB were p=0.1102 and p=0.2482 for 2 days and 10 days extracts, respectively and 2 day vs. 10 days extracts were p=0.1489 and p=0.3865 for 0% and AB, respectively.
